# Alternative splicing: transcriptional regulatory network in agroforestry

**DOI:** 10.3389/fpls.2023.1158965

**Published:** 2023-04-12

**Authors:** Syed Sarfaraz Hussain, Manzar Abbas, Sammar Abbas, Mingke Wei, Ahmed H. El-Sappah, Yuhan Sun, Yun Li, Arthur J. Ragauskas, Quanzi Li

**Affiliations:** ^1^ State Key Laboratory of Tree Genetics and Breeding, Engineering Technology Research Center of Black Locust of National Forestry and Grassland Administration, College of Biological Sciences and Technology, Beijing Forestry University, Beijing, China; ^2^ State Key Laboratory of Tree Genetics and Breeding, Chinese Academy of Forestry, Beijing, China; ^3^ Faculty of Agriculture, Forestry and Food Engineering, Yibin University, Yibin, Sichuan, China; ^4^ College of Biological Sciences and Biotechnology, Beijing Forestry University, Beijing, China; ^5^ Genetics Department, Faculty of Agriculture, Zagazig University, Zagazig, Egypt; ^6^ Department of Forestry, Wildlife, and Fisheries, Center for Renewable Carbon, University of Tennessee Institute of Agriculture, Knoxville, TN, United States; ^7^ Joint Institute for Biological Science, Biosciences Division, Oak Ridge National Laboratory, Oak Ridge, TN, United States; ^8^ Department of Chemical and Biomolecular Engineering, The University of Tennessee-Knoxville, Knoxville, TN, United States

**Keywords:** alternative splicing, transcriptional regulation, intron retaining, gene regulatory network, spliceosome

## Abstract

Alternative splicing (AS) in plants plays a key role in regulating the expression of numerous transcripts from a single gene in a regulatory pathway. Variable concentrations of growth regulatory hormones and external stimuli trigger alternative splicing to switch among different growth stages and adapt to environmental stresses. In the AS phenomenon, a spliceosome causes differential transcriptional modifications in messenger RNA (mRNAs), resulting in partial or complete retention of one or more introns as compared to fully spliced mRNA. Differentially expressed proteins translated from intron-retaining messenger RNA (mRNA^ir^) perform vital functions in the feedback mechanism. At the post-transcriptional level, AS causes the remodeling of transcription factors (TFs) by the addition or deletion of binding domains to activate and/or repress transcription. In this study, we have summarized the specific role of AS in the regulation of gene expression through repression and activation of the transcriptional regulatory network under external stimuli and switch among developmental stages.

## Graphical Abstract

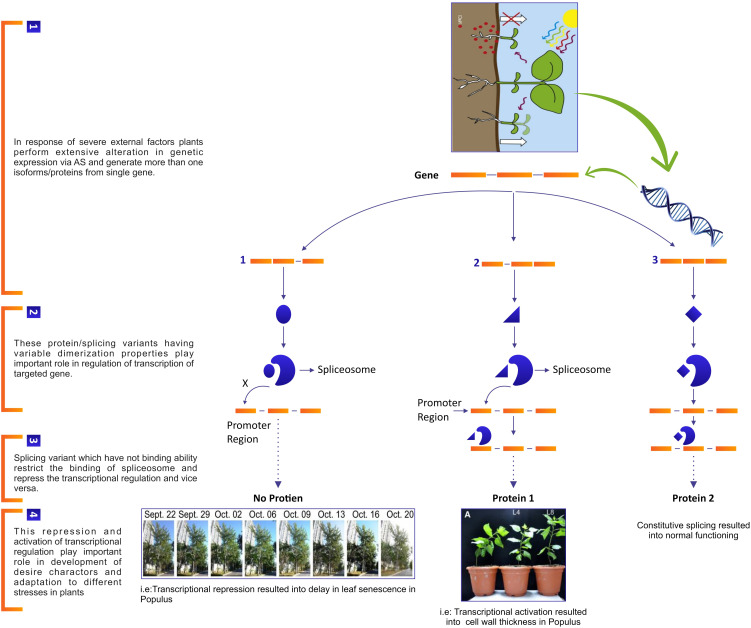

## Introduction

Recent studies showed that alternative splicing (AS) is highly pervasive in plants (Reddy et al., 2013), and introns of more than 60% genes undergo this process (Syed et al., 2012). AS plays a key role in the regulation of gene regulatory networks to help plants adapt to environmental fluctuations (Syed et al., 2012). Protein expression in a specific quantity and at a specific time is crucial for the growth and development of eukaryotic organisms. Protein synthesis is regulated by multiple layers of regulation like cell signaling, assembly of spliceosome and transcription factors, transcription, export of mature mRNA to the cytoplasm, and translation (Thatcher et al., 2016). AS plays a fundamental role in the expression of multiple numbers of mRNA and, subsequently, proteins from fewer genes. In recent studies, it has been shown that AS in multicellular organisms causes significant changes in protein isoforms (Stamm et al., 2005). AS of intron-retaining genes contributes to increasing the coding potential and gene expression regulation through multiple mechanisms such as nonsense-mediated decay (NMD) and microRNA-mediated gene regulation (Reddy et al., 2013; El-Sappah et al., 2021).

AS alters the sequences of functional domains of a protein which shows a different function in a gene regulatory network (Severing et al., 2012). In response to severe external stimuli such as heat, salinity, and shade, plants perform extensive alterations in genetic expression and transcriptome diversity *via* AS to adapt to harsh conditions for their survival (Ding et al., 2020). Kirby (2016) observed that an intense pulsed light treatment of *Arabidopsis thaliana* in the dark stimulates extensive AS to obscure their metabolism. In another study, Tai et al. proved that AS affects lipid biosynthesis in *Picea mariana* by retention of one intron out of four, which resulted in termination in ORF at a premature termination codon (Tai et al., 2007).

Coordination between alternative splicing and transcription is an important phenomenon in the regulation of gene expression (Godoy Herz and Kornblihtt, 2019). A fungal pathogen *Trichoderma* affects the roots of tomatoes which stimulates cytosine methylation, resulting in the activation of AS-regulated differential gene expression (De Palma et al., 2019). Beyond the important role of AS in the increased diversity of proteome by producing novel transcript combinations, presumably, many transcript isoforms have a negative influence over protein structure and function, which suggests its role in the post-transcriptional regulation of gene expression (Campbell et al., 2006). Wang et al. discovered that alternative splicing causes an increased level of snRNA transcripts in pollens which suggests its unique role in the regulation of snRNA biosynthesis in plants (Wang et al., 2020). In this review article, we have summarized recent advances in determining the specific role of AS in the transcriptional regulation of gene expression in plants under the influence of different environmental conditions.

## Mechanism of alternative splicing

**Figure 1:**
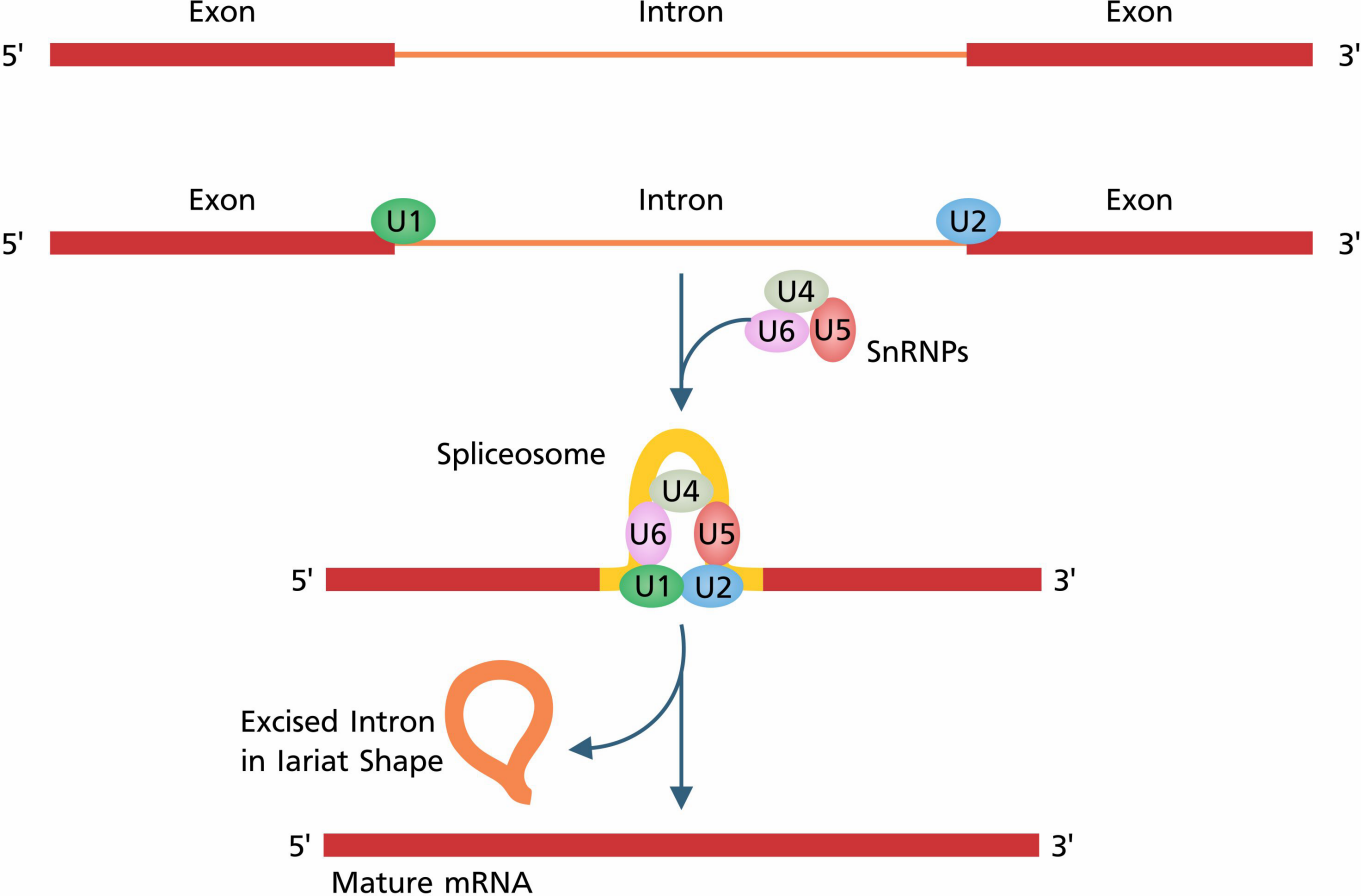
The basic process of constitutive splicing starts with the recognition of splice sites by the spliceosome. U1 and U2 are attached to the 5´ splice site and 3´ splice site, respectively. Splice site recognition is followed by the complete assembly of the spliceosome. After complete assembly of the spliceosome, splicing occurs in two main steps: first, 5´ splice site cleavage and lariat formation, and then, 3´ splice site cleavage and exon ligation.

The initiation of transcription and post-transcriptional modifications are subject to genetic and epigenetic factors. Epigenetic factors cause modifications such as chromatin remodeling, DNA methylation, and RNA-associated gene silencing to switch on/off genetic regulatory elements. Transcription factors (TFs) are comprised of *cis* and *trans* elements (Sperling, 2007) which are recognized by spliceosomes to perform post-transcriptional modifications. Spliceosome is a complex structure that consists of a group of five (U1, U2, U4, U5, and U6) small nuclear ribonucleoproteins (snRNPs) and several auxiliary proteins such as HnRNP proteins, SR proteins, mnRNP proteins, exon junction complex (EJC), and non-snRNP proteins responsible for the splicing of transcripts (Wahl et al., 2009). The assembly of spliceosome and excision of introns consists of the following steps: (i) the recognition of the splicing site by snRNPs (U1, U2, U4, U5, and U6) and other auxiliary factors (Staley and Woolford, 2009), (ii) the assembly of the spliceosome, (iii) the 5´ splice site cleavage and lariat formation, and finally (iv) the 3´ splice site cleavage and exon ligation (**Figure 1**) (Jurica and Moore, 2003). After ligation of exons, all components of the spliceosome are disassembled for the assembly of the next spliceosome complex and the splicing of Pre-mRNA.

The precise selection and inclusion of specific exons in mature mRNA are predominantly controlled by *cis* and *trans* components which determine protein types or proteoforms to perform specific metabolic processes. *Cis* and *trans* factors are comprised of pre-mRNA sequences and protein regulators, respectively. Notably, a clear distinction between splicing factors and elements of constitutive and alternative splicing is under discovery. It is hypothesized that the selection of exon might be influenced by the relative functional strength of splicing sites (Matlin et al., 2005). On the basis of position and function, *cis-*regulatory elements are divided into the following four categories: exonic splicing silencers (ESSs), exonic splicing enhancers (ESEs), intronic splicing silencers (ISSs), and intronic splicing enhancers (ISEs). Recent studies revealed that despite retaining splicing sites, pseudo-exons do not go under splicing and ESSs/ESEs play their role in determining authentic and pseudo-exons (Zhang and Chasin, 2004).

**Figure 2:**
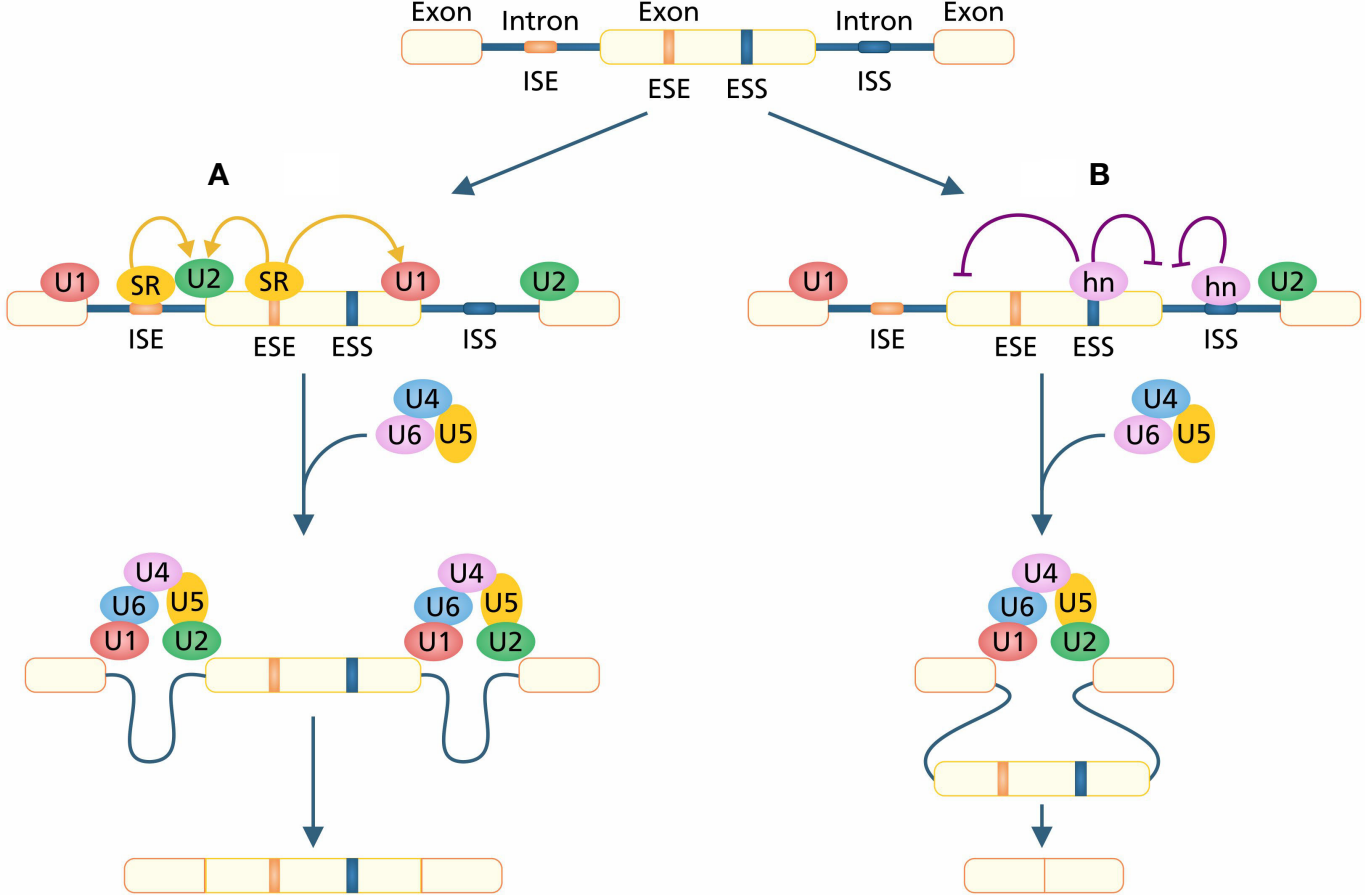
**(A)** The constitutive splicing in which SR proteins are attached on enhancer sites and SR proteins provide sites for the attachment of U1 and U2 on 5´ and 3´ splice sites, respectively. After complete assembly of the spliceosome, introns are excised out and exons ligated to generate mature mRNA. **(B)** Silencers play a role in changing the splice site selection. hnRNPs are attached to silencers, inhibiting the attachment of SR proteins which causes a shift in site selection. A shift in site selection leads to the skipping of exons and the generation of exon-missed mRNA.

The serine and arginine-rich (SR) proteins play a pivotal role in the functioning of both constitutive and regulated splicing (Tacke and Manley, 1999). SR proteins are also involved in post-splicing activities such as mRNA translation, nonsense-mediated mRNA decay, and the export of mRNA to an extranuclear matrix (Long and Caceres, 2009). In pre-mRNA splicing, recognition and recruitment of U1AF (U1 auxiliary factor) splicing factor at 5’ and U2AF splicing factor at 3’ splicing sites is of primary importance, which is also regulated by SR protein *via* protein–protein interaction (Wu and Maniatis, 1993). SR proteins have two functional domains; an N-terminus RNA binding domain which is composed of multiple RNA recognition motifs (RRMs) and an arginine-serine rich C-terminus domain. RRMs initiate sequence-specific RNA binding, and RS domain performs splicing activity (Shen and Green, 2004). ISSS and ESSs are binding sequences of HETEROGENEOUS NUCLEAR RIBONUCLEO PROTEINS (hnRNPs) that bind with RNA by RRM-type and KH-type binding domains (Krecic and Swanson, 1999; Smith and Valcárcel, 2000). The competition between the binding of hnRNPA1 and one of the essential SR protein splicing factors SF2/ASF determines the shift in site selection (**Figure 2**). SF2/ASF regulates binding of U1 whereas hnRNPA1 inhibits binding of U1 snRNP binding on 5´ splicing site (Eperon et al., 2000). ESSs change the splicing site recognition by inhibiting the use of intron-proximal 5´ and 3´ splicing sites. This alteration of splicing site selection leads to the regulation of intron retention in mature mRNA under AS (Wang et al., 2006).

## Alternative splicing and transcriptional regulatory network

**Figure 3:**
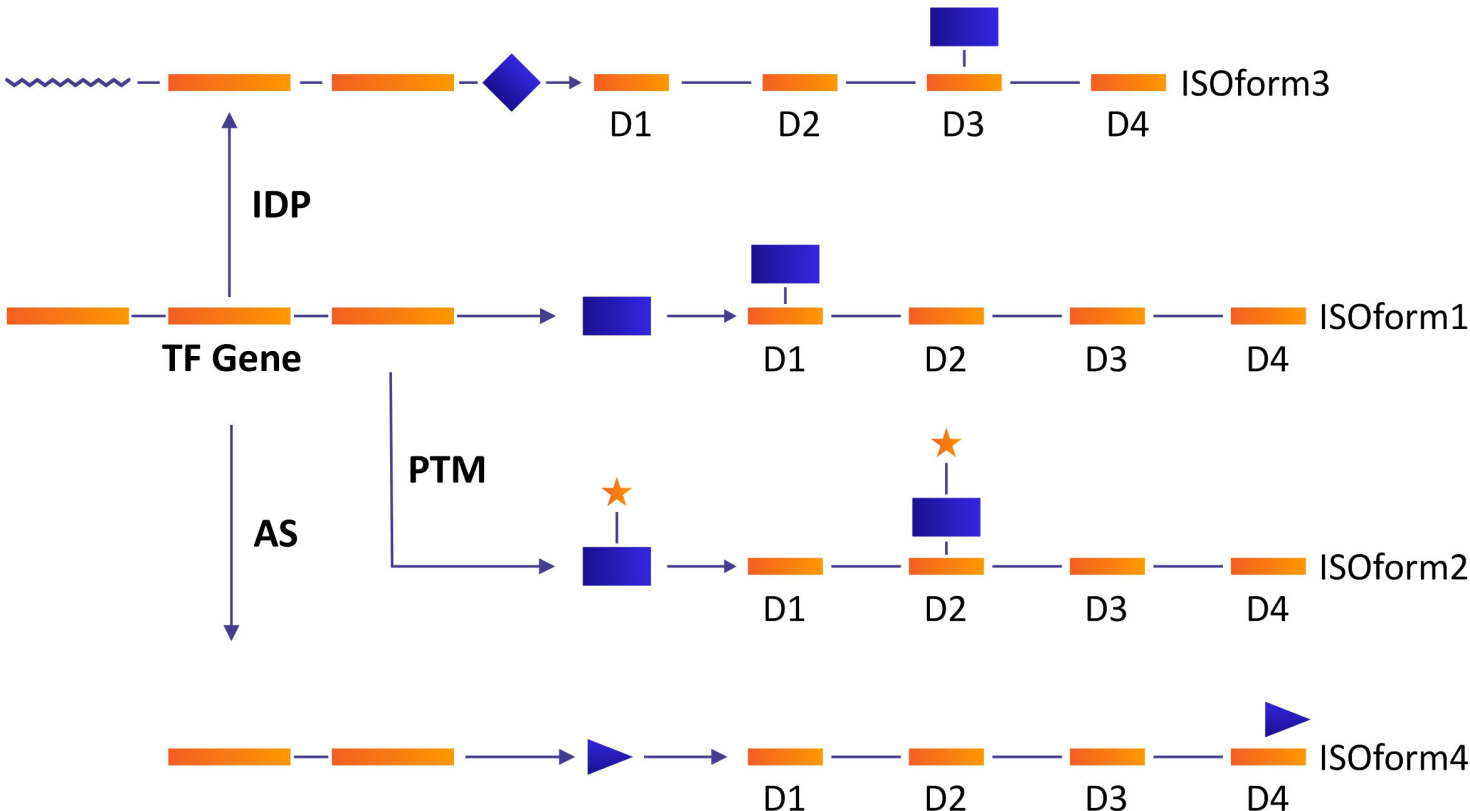
A model of the role of IDP, PTM, and AS in the modulation of gene regulatory network which resulted in modification of protein synthesis. Under normal conditions, transcription factor proteins bind to the promoter of the target gene with extremely high affinity (Isoform 1). The variant as a result of IDPs, PTMs, and AS may change its binding ability and bind to different domains, resulting in a variety of isoforms. A variation in TF protein by PTM (e.g., phosphorylation) may result in the binding ability and production of isoform 2. Isoform 3 may be produced as a result of AS (exclusion of one exon and retention of the intron), and IDP may result in the synthesis of isoform 4.

The Gene regulatory network (GRN) under TFs plays a pivotal role in numerous cellular functions. Certain models of transcriptional regulation have been proposed including context-based binding of multiple transcriptional factors (TFs) and post-transcriptional modifications (PTMs) (Schacht et al., 2014). AS affects the regulation of transcription *via* the inclusion and exclusion of binding domains, a modification in the stability of transcripts, and protein activity (Garcia et al., 2004). TFs harbor intrinsically disordered proteins (IDPs) and precursor RNAs which are subjected to AS. The coeffects of AS, IDPs, and PTMs cause modifications in proteins involved in cell signaling and cell fate, and, consequently, interrupt the gene regulatory network (**Figure 3**) (Niklas et al., 2015). In *Medicago sativa*, the heat shock transcription factor (MsHSF1) is comprised of three introns and four exons which are transcribed into five different isoforms under AS (He et al., 2007). Only MsHSF1b binds with the target gene promoter because all the rest carry premature termination codons and are low-abundance. Isoform MsHSF1b is comprised of the 1st and 4th exons while the other four transcripts retained intervening sequences along with the 1st and 4th exons, which lead to nonsense-mediated mRNA decay (He et al., 2007). Similarly, *PtrSND1-A2*(*WND1B*) is a secondary wall thickening associated with TF which is transcribed into two isoforms *PtrSND1-A2* (*PtrWND1B-s)* and *PtrSND1-A2^ir^* (*PtrWND1B-I*) and both are antagonistic in their function. The overexpression of *PtrWND1B-s* resulted in a thick cell wall, while the overexpression of *PtrWND1B-I* resulted in a thin cell wall (Zhao et al., 2014). So, AS plays a key role in the transcriptional regulatory network *via* repression or activation of TFs.

## Applications of as in transcriptional repression

Dimerization modulates the activities of TFs. Homodimers increase the DNA-binding specificity and affinity of TFs, while heterodimers diversify the affinities and specificities of TFs (Kosugi and Ohashi, 2002). Protein-protein interaction and signal transduction are two important functions of dimerization that create functional diversity in various biological and physical processes by different combinations of proteins having distinct regulatory functions (Klemm et al., 1998). AS is also involved in the generation of splicing variants having variable dimerization properties. A biochemically distinct mechanism of negative regulation is the formation of heterodimers by competitive inhibition of small proteins (siPEP) generated by AS (Seo et al., 2011a).

### AS in wood formation

Programmed cell death and secondary cell wall (SCW) formation are two responsible phenomena for wood formation. Wood, which is a primary source of pulping, energy, and wood products, is composed of a secondary cell wall having major components like lignin, cellulose, and hemicelluloses. NAC-MYB is a major complex of transcriptional factors which is involved in the biosynthesis of the secondary cell wall (Nakano et al., 2015). In *Populus* 6031 and *Eucalyptus* 2987, mRNAs were transcribed by AS, which shows the key role of AS in wood formation (Xu et al., 2014). AS-mediated NAC (for NAM, ATAF1/2, and CUC2) transcription factors regulate the biosynthesis of SCW in *Populus*. PtrSND1-A2 is one of the four family members of Secondary Wall-Associated NAC Domain 1 (SND1) TFs which are involved in the activation of genes associated with cell wall biosynthesis. PtrSND1-A2^ir^ is a splice variant retaining a second intron that lacks in binding domain but can still dimerize. (Li et al., 2012). Reportedly, PtrSND1-A2^ir^ (**Table 1**) represses all members of SND1 and VND6 including Vascular-Related NAC-Domain 6 C1 (PtrVND6-C1) through heterodimerization except PtrSND1-A2 (Lin et al., 2017). Similarly, PtrVND6-C1^ir^, a member of the PtrVND6-C1 family suppresses all members of the PtrSND1 family including PtrSND1-A2 but has no effect on its cognate PtrVND6-C1. This reciprocal regulation within the gene family suggests a high control of alternative splicing in the regulation of wood formation.

**Table 1:** List of introns retaining transcripts involved in transcriptional suppression.

Sr. No	Gene	Isoforms	Host Plant	Reference
1	*PtrSND1-A2 (PtrWND1B)*	*PtrSND1-A2^ir^ (PtrWND1B-l)*	Populus	(Li et al., 2012; Zhao et al., 2014)
2	*PtrVND6-C1*	*PtrVND6-C1^ir^*	Populus	(Lin et al., 2017)
3	*CCA1*	*CCA1β*	Arabidopsis	(Park et al., 2012)
4	*CO*	*Coβ^ir^*	Arabidopsis	(Gil et al., 2017)
5	*IDD14*	*IDD14β^ir^*	Arabidopsis	(Seo et al., 2011b)
6	*OsHSFA2d*	*OsHSFA2dII*	Rice	(Cheng et al., 2015)
7	*FT2*	*FT2ß*	Arabidopsis	(Qin et al., 2017)
8	*PtRD26*	*PrRD26^ir^*	Populus	(Wang et al., 2021)
9	*INH2*	*INH2β*	Potato	(Brummell et al., 2011)
10	*PIF6*	*PIF6-β*	Arabidopsis	(Penfield et al., 2010)
11	*FLM*	*FLM-δ*	Arabidopsis	(Posé et al., 2013)

### AS in cold tolerance

Among environmental stresses, cold is a major stress which affects the growth and development of plants. Plants have developed various strategies and mechanisms to cope with cold stress (Holzhaider et al., 2011). Transcription factors basic-helix-loop-helix (bHLH) play a key role in growth, development, and abiotic stress. In the *Eucalyptus* hybrid (*E. nitens × E. globulus*), 44 bHLH transcripts were transcribed under the effect of AS and two unique isoforms during cold acclimation to counter cold stress (Apablaza et al., 2021). Late Elongated Hypocotyl (LHY) and Circadian Clock-Associated 1 (CCA1) coexpress to regulate the transcription of C-Repeat Binding Factor (CBF) to enhance cold tolerance (Dong et al., 2011). Two splice variants transcribed from CCA1 are full-size CCA1α and truncated CCA1β. CCA1α lacks N-terminus DNA binding domain but retains a protein-protein interaction domain for dimer formation. The splice variant CCA1β regulates the transcription of CCA1 and also makes non-functional CCA1α-CCA1β and LHY-CCA1β heterodimers to suppress CCA1α and LHY. The cold stress inhibits AS and restores transcription to full-size CCA1α to enhance cold tolerance (Park et al., 2012).

### AS regulates flowering time

**Figure 4:**
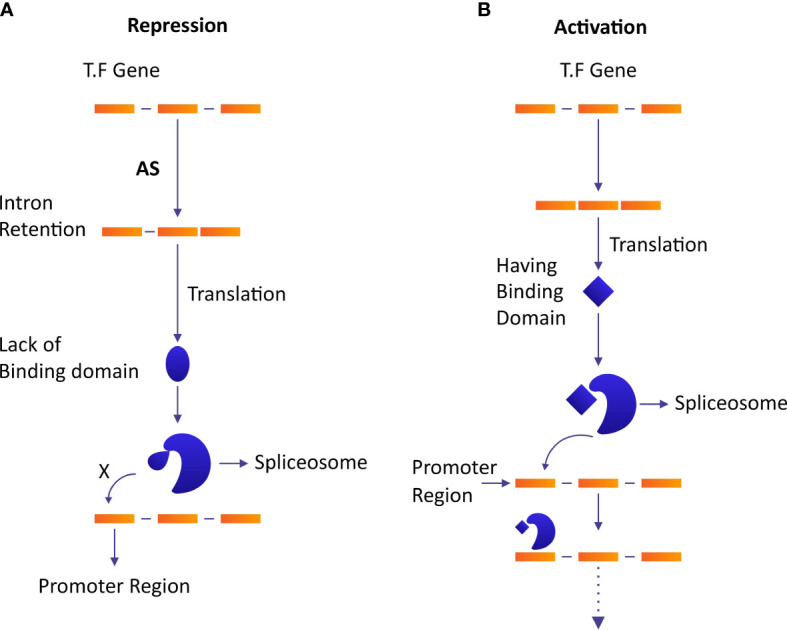
The role of AS in the repression and activation of the transcriptional regulatory network. Homodimers increase the DNA-binding specificity and affinity of TFs, while heterodimers diversify the affinities and specificities of TFs. **(A)** AS causes a modification of the structure of TF due to intron retention and changes its binding ability which results in an inhibition of the binding of spliceosome complex and represses the regulation of gene expression. **(B)** The activation of GTN in absence of AS.

Plants adjust their flowering time in response to numerous environmental and developmental signals under the effect of AS. Among 12688 differentially expressed genes (DEGs) in an early flowering mutant of trifoliate orange (*Poncirus trifoliata*), 16343 AS events were identified among which 3´ splice sites in AS were dominant (Ai et al., 2012). In Arabidopsis, CONSTANS (CO) TFs were involved in the regulation of the expression of floral integrator flowering locus T (FT). CO is transcribed into fully spliced COα and intron retaining COβ^ir^ under the effect of AS. COβ^ir^ isoform lacked C-terminus DNA binding domain which resulted in premature termination. The E3 group of ubiquitin ligases regulates the stability of CO, while osmotically responsive genes 1 (HOS1) and constitutive photomorphogenic 1 (COP1) degrade CO early in the morning and at night, respectively. Whereas, flavin-binding Kelch repeat F-box1 (FKF1) keeps a stable expression of CO in the late afternoon during long days. COβ^ir^ makes heterodimer with COα to inhibit its regular binding with the promoter of CO during the daytime to let COP1 and HOS1 suppress CO. In the afternoon during long days, a relative accumulation of both isoforms increases and FKF1 stabilizes the regulation of COα to overcome Coβ-modulated inhibition (**Figure 4A**) (Gil et al., 2017). This result shows that AS ensures the sustainability of diurnal accumulation dynamics in the photoperiodic flowering of Arabidopsis.

### AS and anthocyanin synthesis

Worldwide, the important ornamental white flower plant Chrysanthemum sometimes produces red color fluorites due to the accumulation of anthocyanin color pigment (Li et al., 2021). Flavonoid biosynthetic genes are regulated by special ternary transcriptional factor complexes of MYB-Basic, helix-loop-helix, and WD40 (MBW) (Xu et al., 2015). It has been revealed that mutation in the promotor region of dihydroflavonol-4-reductase (DFR) and alternative splicing of *DFR* leads to alteration in the accumulation of anthocyanin pigment in the solanum group (Wang et al., 2022). In the chrysanthemum transcriptional factor, *cmbHLH2* generates two transcripts: fully transcribed *cmbHLH2* ^full^ and truncated short-length *cmbHLH2* ^short^. Results showed that short-length alternatively spliced transcript did not have the ability to combine at the promoter region of the *cmDFR* gene, resulting in the failure in activation of the MBW complex and leading to an inactivation in the biosynthesis of the anthocyanin pigment. These results showed that alternative splicing plays the role of a repressor in the pigmentation of anthocyanin (Lim et al., 2021).

### AS regulates starch accumulation

Plant starch is a non-structural carbohydrate that is a major source of carbohydrates during the absence of photosynthesis (Streb and Zeeman, 2012). In *Arabidopsis*, starch metabolism is regulated by INDERMINATE DOMAIN 14 (IDD14) TF (Seo et al., 2011b). The full-length IDD14α transcript binds with the promoter region of the *Qua-Quine Starch* (QQS) gene to regulate starch metabolism by generating competitive inhibitors. Under cold stress, the first intron retaining IDD14β^ir^ is transcribed because of AS, which lacks a DNA-binding domain and makes a heterodimer with IDD14α. The formation of IDD14α-IDD14β^ir^ heterodimer restricts the transcription of the QQS gene and results in starch accumulation. This negative regulation of starch metabolism is a cold stress adaptation strategy to store starch under dark conditions in order to maintain a certain amount of starch to cope with cold stress during day time (Seo et al., 2011b).

### Leaf senescence and alternative splicing

A very complex gene regulatory network comprised of hundreds of regulatory factors is involved in the regulation of leaf senescence in plants (Woo et al., 2019). Leaf senescence is a very important stage of development for plant fitness that is regulated by senescence-associated NAC family transcription factors (Sen-NAC TFs) (Wang et al., 2021). AS of Sen-NAC TFs provides an additional layer of post-transcriptional regulation, which maintains the mechanism of leaf senescence. *PtRD26*, a member of Sen-NAC TFs, is spliced into an intron-retaining variant *PrRD26^ir^* in *Populus tomentosa*. *PtRD26^ir^* negatively regulated the functions of senescence-related TFs by repressing their binding ability, which resulted in the delay in leaf senescence (Wang et al., 2021).

## Applications of as in transcriptional activation

### Heat-induced alternative splicing

In plants, abiotic stress factors cause severe biochemical changes which affect pathways involved in the biosynthesis of proteins. In western poplar, 164 out of 15087 AS-mediated abiotic stress-specific isoforms were identified (Filichkin et al., 2018). Heat shock proteins (*Hsp70* & *Hsp83*) play a pivotal role in heat and heavy metals stress tolerance (El Sappah et al., 2021). Endoplasmic reticulum chaperone *OsBiP1* is an unfolded protein response marker that contributes to protein folding (Foresti et al., 2003). In rice, OsHSFA2d regulates the expression of *OsBiP1* to regulate signaling pathways of unfolded protein and heat shock response which is spliced into two main variants OsHSFA2dI and OsHSFA2dII by retaining 94 and 270 bp introns, respectively. The OsHSFA2dI variant only activates the transcription of *OsBip1* under abiotic stress (Cheng et al., 2015). In Arabidopsis, heat shock factor A2 (*HsfA2*) is a key regulator of heat stress. Under moderate heat stress (37°C), a 31 nt long intron retaining transcript *HsfA2-II^ir^* is transcribed under the effect of AS (**Table 2**). Under severe heat stress (42°C to 45°C), 1a intron retaining *HsfA2-III^ir^* is expressed which activates the transcription of a small truncated isoform S-HsfA2 responsible for the self-regulation of the transcription of HsfA2 (Liu et al., 2013).

**Table 2:** List of introns retaining transcripts involved in transcriptional activation.

Sr. No	Gene	Isoforms	Host Plant	Reference
1	*OsHSFA2d*	*OsHSFA2dI*	Western poplar	(Cheng et al., 2015)
2	*HsfA2*	*HsfA2-III^ir^*	Arabidopsis	(Liu et al., 2013)
3	*MTPs*	*PvMTF-1*	Bean	(Yang et al., 2015)
4	*PIF6*	*PIF6-α*	Arabidopsis	(Penfield et al., 2010)
5	*FLM*	*FLM-β*	Arabidopsis	(Posé et al., 2013)
6	*PtrWND1B*	*PtrWND1B-s*	Populus	(Zhao et al., 2014)

An interesting work by Ling et al., 2018 investigated a heat shock tolerance strategy of Arabidopsis named heat-shock memory. IR alternative splicing plays a key role in the tolerance of heat in Arabidopsis, but a dramatic change by the plant was observed in response to a second exposure of the plant to non-lethal heat shock. Plants exposed to non-lethal heat shock retained a high level of IR and do not respond to the second heat shock which is favorable for the normal growth and development of the plant. This study also emphasizes the important role of AS in the heat tolerance of plants.

### Metal responsive element

Trace metal elements’ stress has serious effects and consequences on plant growth and animal health upon ingestion. Metal tolerance proteins (MTPs) play an important role in heavy metal stress resistance and metal accumulation (El-Sappah et al., 2021; Abbas et al., 2022). AS regulates gene expression *via* intron-specific promoters by increasing the diversity of MTPs transcripts which results in a significant halt in the accumulation of trace metal elements in plant tissues. In bean (*Phaseolus vulgaris*), metal-responsive elements binding the transcription factor *PvMTF-1* regulate the transcription of abiotic stress-responsive gene *PvSR2* (Yang et al., 2015). In this case, an intronic promoter transcribes *PvMTF-1* to complement metal-responsive elements by hybridization at the upstream region of the *PvSR2* promoter to regulate its expression (**Figure 4B**) (Yang et al., 2015).

### Alternative splicing of Serine/Arginine-rich proteins

It has been noticed that SR proteins play an important role in the splicing of mRNA and regulation of alternative splicing (Long and Caceres, 2009). Zhao et al. (2021) studied 24 SR genes of *populus trichocarpa* and found that these 24 *PtSR* genes could undergo alternative splicing and generate 45 transcripts under abiotic and hormone stresses. Among these 24 *PtSR* genes, the overexpression of *PtSCL30* negatively regulates the cold and salt stress-responsive genes by making changes in the AS of these genes. In Arabidopsis, 95 transcripts of SR genes were transcribed from 15 genes under the effect of AS (Palusa et al., 2007). Heat stress stimulates AS of several SR genes while hormone stress stimulates AS of only three SR genes (Wang et al., 2021). Protein sequencing analysis revealed that truncated functional domains with distinct functions in different polypeptide isoforms were produced under the effect of AS (Palusa et al., 2007). SR1 gene in *Arabidopsis* is transcribed into five different isoforms out of which only one is fully spliced. SR1, SR1B, and SR1C were transcribed by targeting alternative 3´ splice sites, and the remaining two SR1D and SR1E were transcribed by targeting 5´ splice sites of intron 9. The higher ratio of SR1B/SR1 under high temperatures revealed the crucial role of SR1B in adaptation to heat stress (Lazar and Goodman, 2000).

## Future prospects

AS emerges as a source of diversity in gene expression to cope with biotic and abiotic stresses. Under AS, numerous transcripts are spliced from a single transcript due to (i) choices of alternative site selection by the spliceosome, (ii) intron retention, (iii) selection of alternate exons, and (iv) premature stop codons. In this study, we summarized the interaction of different abiotic stress factors with AS and invited further studies to investigate the signaling pathways involved. Over time, the global environment is adversely changing and is affecting the weather pattern. This change in the climate is having a bad effect on plant growth and all other forms of life. These insights will be helpful in better understanding the adaptation of plants to environmental stresses and the evolution of multicellular organisms. Furthermore, it will be helpful to improve the withholding capacity of plants under different environmental stresses. We also summarized the specific roles of AS involved in the transcriptional regulatory network. Notably, TFs and SR proteins are involved in the constitutive and alternative splicing of different transcripts. Further deep insights into AS of SR proteins in different plant species and the effects of their isoforms on the splicing of mRNAs will open new avenues in understanding the diversity of gene expression. Deep insights into AS involved in cell wall biosynthesis will provide a precise benchmark for the genetic modification of ligno-cellulosic contents in wood.

## Conclusion

The increase in the number of genes as a result of alternative splicing makes it more important to understand its mechanism and function in regulation. Numerous examples of AS responding to various stresses like heat, cold, hormones, and heavy metals have been observed in plants, which plays an important role in the adaptation by producing diverse forms of proteins. AS also diversifies protein production by the suppression and activation of the transcriptional regulation of genes and the splicing of SR genes. There is a need to explore events and functions of AS in plants, which can be helpful for the development of desired growth characteristics and adaptation to different stresses in plants.

## Author contributions

Conceptualization: SH, MA, and QL; Writing-original draft and drawing figures: SH, MA, SA, YS, YL, and AE-S; Editing and proof reading: AR, YL, and QL; Writing the final manuscript: SH and MA. All authors reviewed and approved the final submission.
